# Distribution patterns of haplotypes for symbionts from *Umbilicaria esculenta* and *U. muehlenbergii* reflect the importance of reproductive strategy in shaping population genetic structure

**DOI:** 10.1186/s12866-015-0527-0

**Published:** 2015-10-15

**Authors:** Shunan Cao, Fang Zhang, Chuanpeng Liu, Zhihua Hao, Yuan Tian, Lingxiang Zhu, Qiming Zhou

**Affiliations:** SOA Key Laboratory for Polar Science, Polar Research Institute of China, No.451 JinQiao Road, Pudong District, Shanghai, 200136 China; School of Life Science and Technology, Harbin Institute of Technology, 2 Yikuang Street, Harbin, 150080 China; Institute of Agricultural Product Quality Standard and Testing Research, Tibet Academy of Agricultural and Animal Husbandry Sciences, Lhasa, 850032 China; College of Engineering Management, Inner Mongolia Technical College of Construction, Huimin District, Hohhot, 010070 China; CAS Key Laboratory of Genome Sciences and Information, Beijing Institute of Genomics, NO.1 Beichen West Road, Beijing, 100101 China; Institute of Microbiology, Chinese Academy of Sciences, No.1-3, Beichen West Road, Beijing, 100101 China

**Keywords:** AMOVA, Haplotype, Lichen, Mycobiont, Photobiont, Phylogenetic analysis

## Abstract

**Background:**

The diversity of lichen fungal components and their photosynthetic partners reflects both ecological and evolutionary factors. In present study, molecular investigations of the internal transcribed spacer of the nuclear ribosomal DNA (ITS nrDNA) region were conducted to analyze the genetic diversity of *Umbilicaria esculenta* and *U. muehlenbergii* together with their associated green algae.

**Result:**

It was here demonstrated that the reproductive strategy is a principal reason for fungal selectivity to algae. *U. muehlenbergii,* which disperses via sexual spores, exhibits lower selectivity to its photosynthetic partners than *U. esculenta*, which has a vegetative reproductive strategy. The difference of genotypic diversity (both fungal and algal) between these two *Umbilicaria* species is low, although their nucleotide diversity can vary greatly.

**Conclusions:**

The present study illustrates that lichen-forming fungi with sexual reproductive strategies are less selective with respect to their photobionts; and reveals that both sexual and vegetative reproduction allow lichens to generate similar amounts of diversity to adapt to the environments. The current study will be helpful for elucidating how lichens with different reproductive strategies adapt to changing environments.

**Electronic supplementary material:**

The online version of this article (doi:10.1186/s12866-015-0527-0) contains supplementary material, which is available to authorized users.

## Background

Lichens are intimate and long-lived symbioses between photobionts (green alga or cyanobacteria) and mycobionts (lichen-forming fungi). It has been reported that the number of lichen species is over 17,500 [1]. However, only 200 photobionts have been described on the species level based on morphology, with about 100 species of cyanobacteria, and 100 species of green algae [2]. This indicates that some photobiont species must be shared by a wide variety of lichens. Within lichen communities, there seems to be a photobiont pool that allows different lichen species to share their photobionts [3]. The same photobiont can occur in different lichen species, genera, and families, so they lack value in lichen systematics [4]. However, some lichen-forming fungi exhibit various degrees of selectivity to different photobionts, and they can form lichen thalli with more than one algae species [3, 5].

Lichens whose fungal component reproduces sexually undergo a process of re-lichenization, which means that the mycobiont can exchange its photosynthetic partner for another compatible photobiont in a new lichen thallus [6]. Studies on the relationship between mycobionts and their photobiont partners have increased during the last decade [7–11]. However, most of them do not address lichen reproductive strategies. Generally, selectivity is regarded as the taxonomic range of partners that can be selected by one symbiont, and specificity refers to the degree of selectivity of both partners, which means that the term “specificity” is used for the symbiotic association as a whole [10, 12–15]. In symbioses with sexual reproductive strategies, hosts usually form symbiotic associations with a wide range of partners [16]; so lichens with sexual reproductive structures may exhibit less selectivity toward their photobionts than those with vegetative reproductive structures. It is a great advantage for mycobionts that disperse independently, allowing them to form lichens with locally adapted photobionts [17]. This enhances the possibility of re-lichenization. However, an investigation of algal and fungal phylogenies from lichen genus *Lepraria* suggested that even in lichens dispersed by vegetative structures, the fungi still switch algal partners [18]. Regardless, fungal selectivity toward algal partners and genetic structures of symbionts can be influenced by ecological factors such as microhabitats [9, 19].

Lichen thalli are generally composed of one kind of mycobiont and one type of photobiont. Algal switching may occur when one thallus harbors multiple algal genotypes simultaneously [5, 20, 21]. However, those comprised of multiple symbionts could also be regarded as results of the fusion of adjacent thalli [22]. On many cases, the lichen thallus structure was poorly defined and it is difficult to distinguish the boundaries between individuals. Such lichens are not proper for investigating the genetic structures of symbionts or the relationship between mycobionts and their partners. There is a unique umbilicus (a navel) attached to substrate and a single leaf-shaped thallus in all lichen species belonging to the genus *Umbilicaria*, which ensures that each individual can be distinguished clearly. *Umbilicaria* lichens include the species with vegetative propagules lobes or isidia as their dispersal method, such as *U. esculenta*, and the species with sexual structure ascomata, such as *U. muehlenbergii*. So the species from *Umbilicaria* with different reproductive strategies are ideal for the study of the relationships among symbionts’ dispersal pattern and the diversities of symbionts.

Ribosomal internal transcribed spacer (ITS) is the most sequenced region for fungi [23]. It is often used to reveal the phylogenetic relationship at the interspecies or intraspecies level. This region has been accepted as the best candidate DNA barcoding for most fungi [24, 25]. In addition, ITS also appears to be a promising DNA-barcoding marker in green algae [26, 27]. This sequence has been the one of the most useful sequences in the assessment of the genetic structure of symbiotic partners in lichens [28, 29]. ITS region is widely used to characterize symbiotic interactions between mycobionts and photobionts; for example, the comparison of ITS rDNA phylogenies of symbionts showed that algal switching took place repeatedly among 33 lichens [30], and demonstrated that the range of algal partners from the lichen genus *Physcia* and *Rinodina* was variable [31].

In the present study, genetic variation in the ITS region was investigated for symbionts from *Umbilicaria esculenta* which disperses by vegetative structures and *U. muehlenbergii* in which fungal reproduction is sexual, in order to determine the coupled patterns between each mycobiont and its corresponding photobiont. This work describes the relationship between photobiont flexibility and reproductive strategy and provides a basic account of fungal selectivity for its photobiont partners.

## Methods

### Materials

**Fig. 1:**
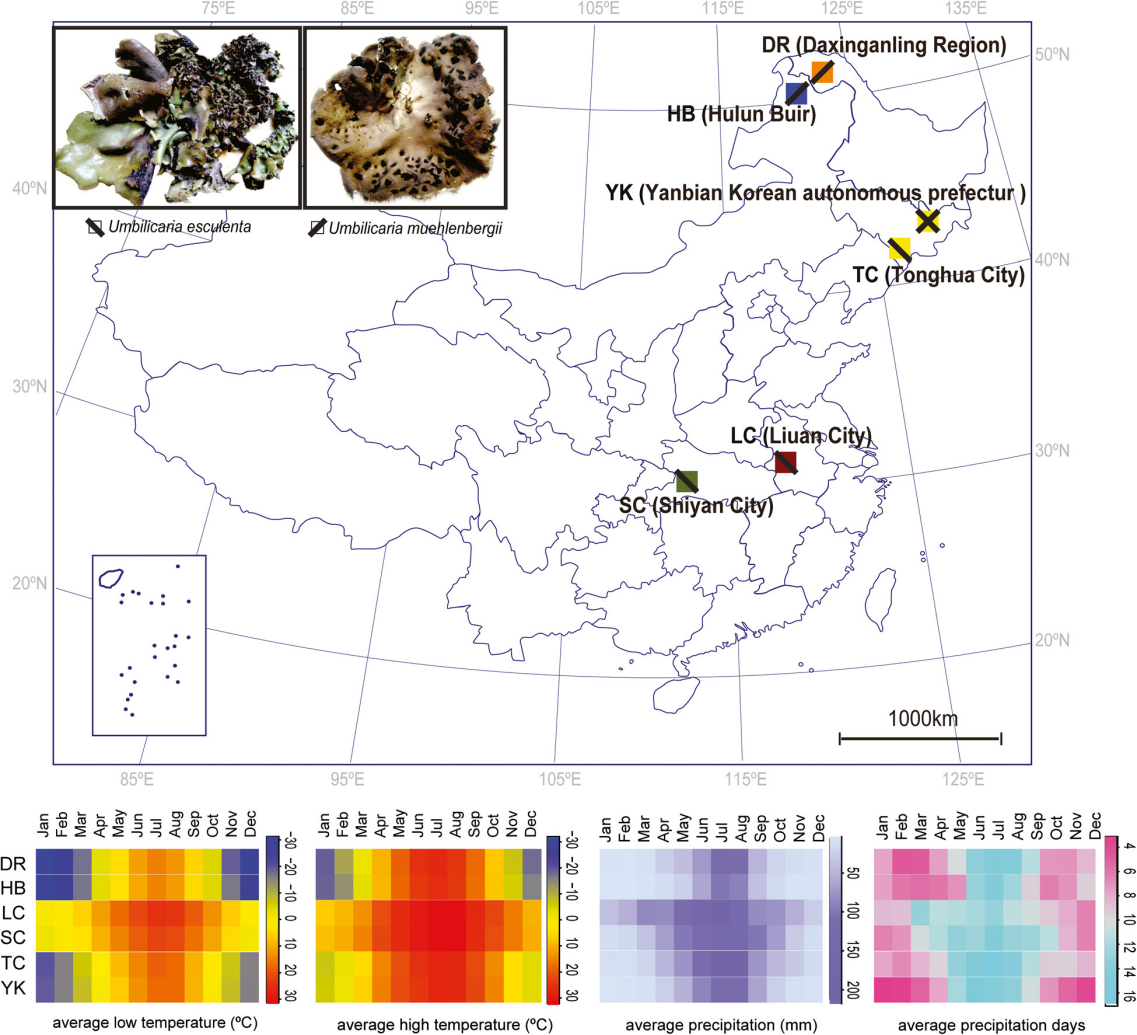
Sketch maps of collecting sites. Samples were collected from six localities in China, and the distribution of *Umbilicaria esculenta* and *U. muehlenbergii* are marked with slash characters (upper). The major climate data for average monthly low temperature, average monthly high temperature, average monthly precipitation, and average precipitation days for these six sampling sites were obtained from Wikipedia, and the corresponding heat maps were constructed using R language (lower)

A total of 172 samples belonging to eight species from six lichen genera were collected from six sampling areas, all within China (Fig. 1). Among the samples, 99 *Umbilicaria esculenta* (Miyoshi) Minks individuals were collected from Yanbian Korean autonomous prefecture (YK), Tonghua City (TC), Shiyan City (SC), and Liuan City (LC), and 63 *U. muehlenbergii* (Ach.) Tuck. individuals were sampled from Yanbian Korean autonomous prefecture (YK), Tonghua City (TC), HulunBuir (HB), and Daxing’anling Region (DR). Samples of *Candelariella coralliza* (Nyl.) H. Magn., *Candelaria fibrosa* (Fr.) Müll. Arg., *Parmelia omphalodes* (L.) Ach., *Parmelia squarrosa* Hale, *Rhizoplaca subdiscrepans* (Nyl.) R. Sant., and *Xanthoparmelia conspersa* (Ach.) Hale. collected in Yanbian Korean autonomous prefecture (YK) where both *U. esculenta* and *U. muehlenbergii* were distributed, were also used in the present study (Additional file 1: Table S1).

### DNA extraction and PCR

Total DNA from mycobionts and photobionts was extracted using a modified CTAB method [32]. The mycobiont ITS sequences were amplified using the fungal specific primer pairs ITS5 and ITS4 [33], ITS1 and ITS4 [33], or ITS1F and ITS4 [34]. The photobiont ITS segments were amplified using the algal specific primer pairs nrSSU-1780 and nrLSU-0012 [30], Al1500bf and LR3 [31], and ITS1T and ITS4T, which are specific to *Trebouxia* species (Additional file 1: Table S3).

The PCR reaction (total volume 50 μL) consisted of 5 μL amplification buffer (contain 25 mmol l^−1^ of MgCl_2_), 1.25 units of *Taq* DNA polymerase (TaKaRa Biotechnology Co. Ltd.), 4 μL 2.5 mmol l^−1^ of each dNTP, 2 μL 10 μmol l^−1^ of each primer, 6 μL of diluted template DNA, and 33 μL H_2_O. The PCR amplification conditions were as follows: initial denaturation at 95 °C for 5 min, followed by 30 cycles of 94 °C for 40 s, 50–55 °C for 40 s, and 72 °C for 2–4 min. These cycles were followed by a final extension at 72 °C for 10 min.

The amplification products were verified electrophoretically in 0.8 % agarose gel and purified with a Gel Extraction Mini Kit (Omega Bio-tek, Inc).

Sequencing reactions were performed with an ABI3730XL Sequencer and double-stranded PCR products were sequenced. The sequencing primers were the same as those used for PCR.

### Data analysis

Double-directional sequences data were checked and assembled using the SEQMAN program within the Lasergene v7.1 software package (DNASTAR Inc.). The regions of the small subunit and large subunit of rDNA flanking the ITS region were trimmed off.

Preliminary alignment of the sequences obtained in the present study and those retrieved from GenBank (Additional file 1: Table S2) was performed using the ClustalW algorithm included in MEGA 5 and then adjusted manually [35]. The best-fit substitution model for each single alignment was calculated using MEGA 5. For the mycobiont and the photobiont data sets, the best-fit models selected using BIC (Bayesian Information Criterion) were K2 + G + I and K2 + G, respectively. The phylogenetic structure of each alignment was constructed with a maximum likelihood (ML) method under the best-fit mode estimated above. The reliability of the inferred trees was tested using bootstrap searches of 1000 resamplings. A SpeciesIdentifier 1.7.7 software tool was used to distinguish the lineages of photobionts [36]. The default parameters were used in all the analyses above.

### Genetic diversity of ITS rDNA

The genetic variety of ITS rDNA sequences was analyzed by AMOVA, which was included with Arlequin 3.5 [37]. ITS sequences of photobionts were divided into three datasets, they were from the photobionts from *U. esculenta*, the photobionts from *U. muehlenbergii*, and those from *U. muehlenbergii* but shared by *U. esculenta*. The sequences of photobionts from the same locality were treated as a population for each data set. The significance was tested with 1000 permutations. The level of polymorphism for both the mycobiont and photobiont of each species in *Umbilicaria* was estimated with the number of polymorphic sites, the haplotype diversity and nucleotide diversity [38]. The haplotype network of the algae was calculated by TCS version 2.1 [39] using the neat ITS sequence data. The parameters used to calculate 95 % parsimony probability haplotype networks by TCS were as follows: the connection limit was at 20 mutational steps and gaps were treated as the missing character state. The figures were prepared in Adobe Illustrator CS4.0 (Adobe Systems).

## Results

A total of 344 sequences from 172 lichen individuals sampling from six locations in China (Fig. 1) were obtained in this study. Mycobiont and photobiont ITS rDNA sequences were recovered from all samples.

The different haplotypes observed were shown in the Additional file 1: Table S1. Four haplotypes were obtained for mycobionts from *U. esculenta* and two from *U. muehlenbergii*. Sequences of the same species showed only a few differences among distinguishable haplotypes (10 sites among four *U. esculenta* haplotypes and one site between two *U. muehlenbergii* haplotypes). All *Umbilicaria* individuals were found to be associated with photobiont of the genus *Trebouxia*. Only one phylogenetic lineage of *Trebouxia jamesii* was found in *U. esculenta* as the photosynthetic partner. However, *U. muehlenbergii* associated with algae belonging to four clades, one of which was shared with *U. esculenta*.

### Mycobiont ITS rDNA sequence analysis

**Fig. 2:**
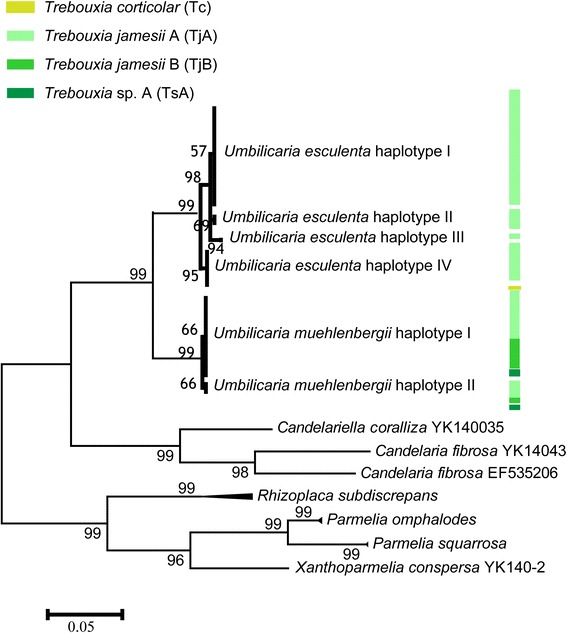
The Maximum Likelihood (ML) tree of mycobiont based on the ITS rDNA sequences. The reliability of the inferred tree was tested by 1000 bootstrap replications, and numbers at nodes present the bootstrap support value (numbers <50 not shown)

The phylogenetic analysis of mycobiont ITS rDNA region was inferred from 172 lichen individuals (Additional file 1: Table S1), and the phylogenetic topology was found to be consistent with previous studies of morphological species (Fig. 2).

*U. esculenta* showed four haplotypes. The most abundant was UeI, of which 76.8 % were sampled from YK, and 23.2 % from SC. The two ITS haplotypes of the total 63 *U. muehlenbergii* individuals were detected, and only one nucleotide differed between them.

### Photobiont ITS rDNA sequence analysis

All the photobiont ITS rDNA sequences amplified from 172 lichen thalli were sequenced (Additional file 1: Table S1). Sequences from photobionts of *Boreoplaca ultrafrigida*, which were also from YK (GenBank Nos. HQ026148-HQ026193, HQ026196-HQ026219) and other related photobiont ITS rDNA sequences retrieved from GenBank (Additional file 1: Table S2), were appended in the phylogenetic analyses.

**Fig. 3:**
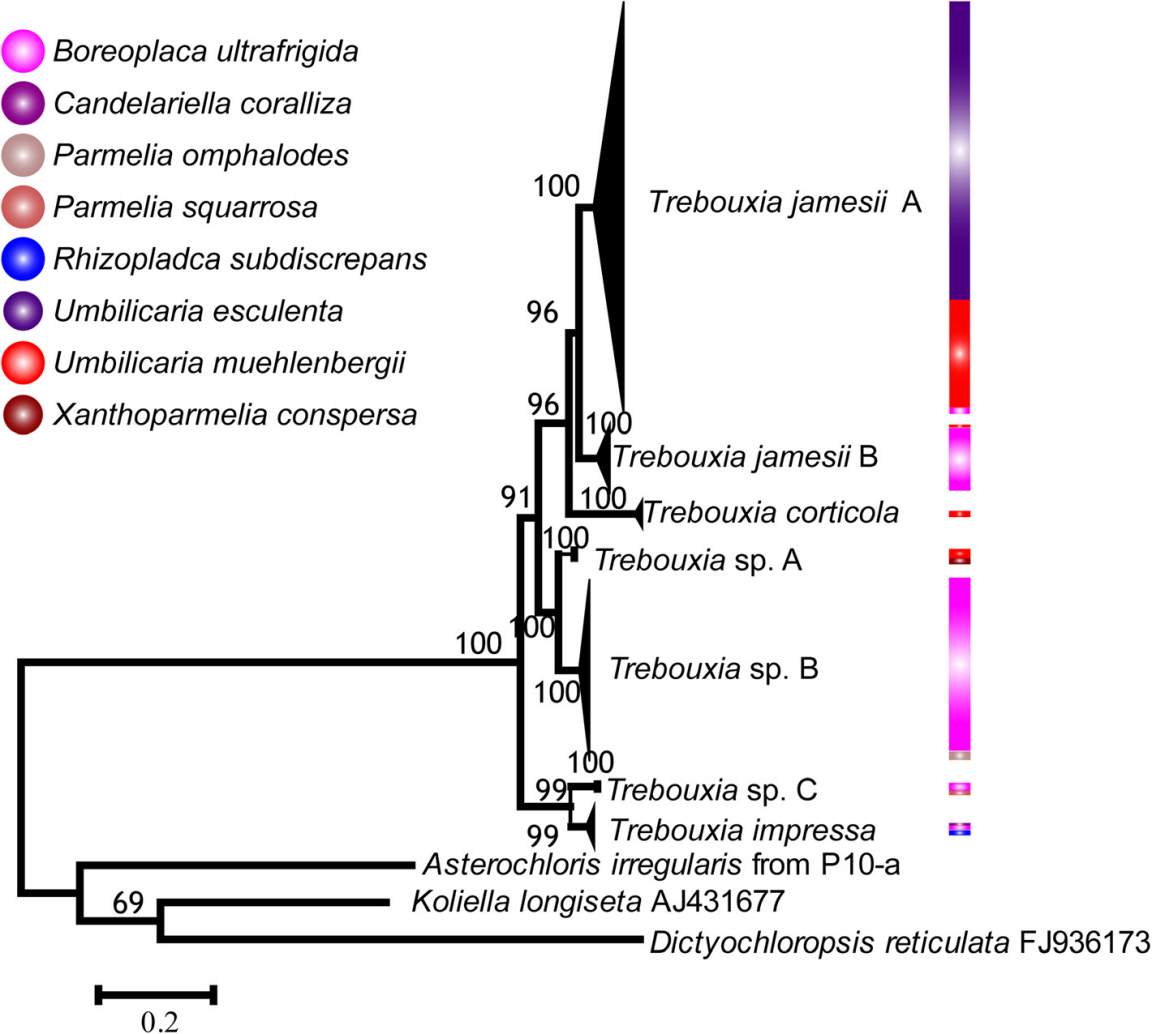
The Maximum Likelihood (ML) tree of photobiont based on the ITS rDNA sequences. The reliability of the inferred tree was tested by 1000 bootstrap replications, and numbers at nodes present the bootstrap support value (numbers <50 not shown). Numerous photobionts classified in the green algae genus *Trebouxia* are divided into different *Trebouxia* species groups. The unidentified lineage are presented with *Trebouxia* sp. Different colors represent different mycobiont or photobiont groups

The results demonstrated that all the photobiont partners inferred in this research were classified in the green algae genus *Trebouxia* de Puymaly (Fig. 3). Phylogeny also resolved seven well supported monophyletic groups and a barcoding analysis using Species Identifier indicated that they could be recognized as distinguishable taxons at species level. Among these photobionts clades, 5.5 % was a threshold to distinguish different species, so the traditional *T. jamesii* was treated as two separate species (Additional file 2: Figure S1). For some clades, species were identified by name, but there were also three unidentified lineages, here called *Trebouxia* spp. The photobiont clades in this study were considered separate species and were represented as follows: *T. corticola* (Tc), *T. impressa* (Ti), *T. jamesii* group A (TjA), *T. jamesii* group B (TjB), *Trebouxia* sp. group A (TsA), *Trebouxia* sp. group B (TsB), and *Trebouxia* sp. group C (TsC). A total of 48 unique photobiont ITS haplotypes distributed in these clades were identified in this study.

The most common photobiont group was TjA, which comprised 137 samples and 22 ITS haplotypes. The haplotypes within TjA were found in *U. esculenta*, *U. muehlenbergii*, and *B. ultrafrigida*. The next most frequent photobiont group TsB was shared by *B. ultrafrigida* and *Parmelia omphalodes*, which comprised 63 samples and six ITS haplotypes. All ITS haplotypes within TsB were found in *B. ultrafrigida* and only haplotype TsB1 was found in *P. ompholodes*. Next, TjB was to be the most common photobiont group. There were 24 samples from *U. muehlenbergii* and *B. ultrafrigida*. They were divided into eight ITS haplotypes but no ITS haplotype was shared by these two species. Among the remaining 23 photobiont samples, 10 distinct ITS haplotypes were detected in four *Trebouxia* groups, Tc, Ti, TsA, and TsC; all of them except for TsA was found to be associated with more than one lichen-forming fungi species, and the Tc was shared by *U. muehlenbergii* and *Xanthoparmelia conspersa*.

**Fig. 4:**
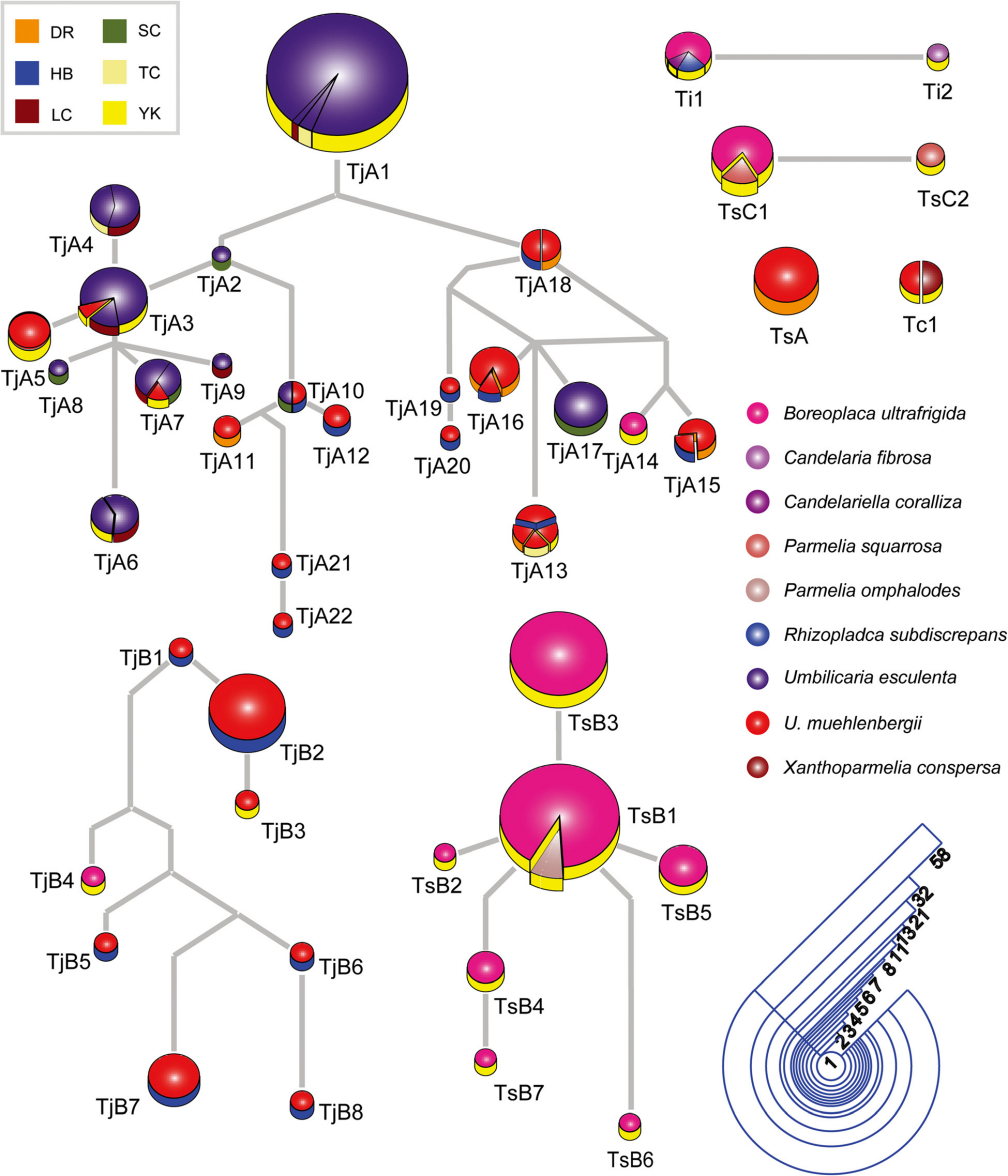
The haplotype network of photobionts. The result was calculated by TCS (95 % parsimony probability, the connection limit of 20 mutational steps and gaps treated as the fifth character state) and decorated using Adobe Illustrator CS4.0. Each circle represents a certain photobiont haplotype, and the size of the pie chart is proportional to the numbers of the samples. Different color of pie charts stands for different lichen species; and in each pie chart, the side surface with different color marks for the collection locations, which are abbreviated as two capitals with the same means as those in Fig. 1

The haplotype networks showed results similar to those of phylogenetic analyses and provided a visual perspective (Fig. 4). The most common photobiont ITS haplotype was TjA1, which comprised 58 *U. esculenta* samples originating from LC, TC, and YK. The second most common ITS haplotype was TsB1, containing 29 *B. ultrafrigida* samples from YK and with three *Parmelia omphalodes* from the same region. Some photobiont haplotypes were shared by different lichen species. For example, Tc1, Ti1, TsB1, and TsC1 were found in *B. ultrafrigida* or *U. muehlenbergii* and other lichen species; TjA3, TjA7, and TjA10 were detected in both *U. esculenta* and *U. muehlenbergii*.

The algal haplotypes exhibiting a multiple localities distribution were all from TjA.

### Analysis of genotypes for lichen species

All *U. esculenta* individuals belonging to four mycobiont ITS haplotypes were associated with TjA as their photobiont, and the most common fungal and algal ITS haplotypes were UeI and TjA1, respectively. There were a total of 19 genotypes (haplotype combination of both mycobiont and photobiont) from *U. esculenta* thalli (Additional file 2: Figure S2a). The most abundant genotype was UeI-TjA1, which was distributed mainly among individuals from YK; and the second most abundant genotype was UeIV-TjA3, which was also distributed among those samples from YK. However, these two genotypes existed in LC, too. The next common genotypes were UeI-TjA17 and UeII-TjA1, and then were genotypes UeIV-TjA1 and UeIV-TjA7, UeI-TjA4, and UeI-TjA6 each contained more than three individuals. Only one or two individuals were found in the remaining genotypes. Among all these genotypes UeI-TjA1, UeI-TjA4, UeI-TjA6, UeII-TjA1, and UeIV-TjA3 were detected in more than one area.

Two mycobiont ITS haplotypes were found in *U. muehlenbergii.* However, its photobionts belonged to 23 different ITS haplotypes, and these could unambiguously be assigned to four groups within *Trebouxia*. A total of 24 genotypes from *U. muehlenbergii* thalli were found. The most abundant genotype was UmI-TjB2. It included 10 individuals. There was only one individual found for each of 12uniquegenotypes (Additional file 2: Figure S2b). The lichen thalli with genotype UmI-TjA13 were found in four localities, and the thalli with genotypes UmI-TjA15, UmI-TjA16, and UmI-TjA18 were collected from two localities.

### Spatial distribution of photobionts

Three data sets of ITS rDNA sequences for photobionts were used to investigate the extent to which geographic factors influence photobiont variation in two *Umbilicaria* species that have different reproductive strategies. In the first two data sets, sequences from all the photobionts of *U. esculenta* or *U. muehlenbergii* were included; and in the third set, only sequences from photobionts from TjA which was the only photosynthetic partner of *U. esculenta*, was selected for *U. muehlenbergii*. In each data set, photobionts from the same locality were treated as a population, and the photobionts with the same haplotype were grouped together within every locality.

The AMOVA results showed that genetic variation of photobionts from *U. esculenta* among localities accounted for 46.98 % of all variation. This value was only 18.00 % for *U. muehlenbergii* (Table 1). Even if TjA, which was the only algal species in *U. esculenta* was considered for *U. muehlenbergii*, the relative amount of variation of photobionts among localities was 17.79 %. The relative amount of variation within localities for the photobionts of *U. muehlenbergii* was 82.00 % (all algal species) or 82.21 % (only TjA), much higher than that for algae of *U. esculenta*.

**Table 1:** AMOVA analysis of photobionts based on algal ITS rDNA

Photobiont				
Source of variation	df.	Sum of squares	Variance components	Percentage of variation
All photobionts (TjA) from *U. esculenta*				
Among localities	3	10.25	0.19 Va	46.98
Within localities	95	20.69	0.22 Vb	53.02
Total	98	30.94	0.41	
Fixation Index	FST:	0.47		
All photobionts from *U. muehlenbergii*				
Among localities	3	4.74	0.09 Va	18.00
Within localities	59	24.19	0.41 Vb	82.00
Total	62	28.94	0.50	
Fixation Index	FST:	0.18		
TjA from *U. muehlenbergii*				
Among localities	3	3.30	0.09 Va	17.79
Within localities	32	12.67	0.40 Vb	82.21
Total	35	15.97	0.48	
Fixation Index	FST:	0.18		

### Molecular diversity of mycobionts, photobionts, and mycobiont-photobiont combinations from *Umbilicaria*

Nucleotide diversity was computed in Arlequin 3.5. It was here treated as equivalent to the gene diversity at the nucleotide level for DNA data.

Among the two mycobionts in *Umbilicaria* species, *U. esculenta* had more haplotypes (four) and polymorphic sites (10) and more nucleotide diversity (0.0049), but only two haplotypes were found in *U. muehlenbergii* and it showed much less nucleotide diversity (Table 2). However, the photobiont from *U. muehlenbergii* had more haplotype diversity (0.93) than that from *U. esculenta* (0.63). The nucleotide diversity of photobiont ITS rDNA from *Umbilicaria* exhibited significant differences among species with sexual reproductive structures (*U. muehlenbergii*) and those with vegetative reproducing structures (*U. esculenta*). When the whole lichen thallus (including mycobiont and photobiont) were considered, the variances of gene diversities between these two *Umbilicaria* species were reduced although the nucleotide diversity was significantly less pronounced in *U. esculenta*.

**Table 2:** Molecular diversity of mycobionts, photobionts and mycobiont-photobiont combinations based on ITS rDNA data

Population	Sequence Number	Haplotype Number	Haplotype diversity	Polymorphic sites	Nucleotide diversity (π)
Mycobiont					
*U. esculenta*	99	4	0.52 +/− 0.04	10	0.0049 +/− 0.0029
*U. muehlenbergii*	63	2	0.23 +/− 0.06	1	0.0005 +/− 0.0006
Photobiont					
From *U. esculenta*	99	10	0.63 +/− 0.05	23	0.0050 +/− 0.0029
From *U. muehlenbergii*	63	23	0.93 +/− 0.01	161	0.0596 +/− 0.0292
Myco-photobiont					
For *U. esculenta*	99	19	0.79 +/− 0.04	33	0.0049 +/− 0.0027
For *U. muehlenbergii*	63	24	0.95 +/− 0.01	162	0.0331 +/− 0.0162

## Discussion

Though some studies have shown that chemicals such as lectins may play an important role in the recognition and association between mycobionts and photobionts, the mechanisms by which lichen-forming fungi select specific photobiont partners are still poorly understood [40]. It has been suggested that many ecological and evolutionary factors, such as fungal and algal dispersal, mode of algal transmission, availability of compatible photobiont partners, adaptation of photobiont and mycobiont to local environmental conditions, and substrate, are main determinant of the association between mycobionts and photobionts. It is therefore difficult to predict the patterns of photobionts’ genetic structures in lichens [19, 41].

Many lichens disperse via vegetative reproduction under most circumstances but occasionally produce ascomata. For this reason, it is difficult to affirm that a lichen species distributes solely via vegetative reproduction. However, a recent study on the mixed species *Physconia grisea* and related sexual species *P. distorta* demonstrated that although both species produce mature apothecia and discharge meiospores capable of germination when cultured, very few (0.43 %) spores from *P. grisea* developed while spores from *P. distorta* were able to develop successfully [42]. It indicated that the ascomata in mixed species lacked full reproductive function and that vegetative propagation was significantly dominant in these lichens. For this reason, *U. esculenta* was considered a strictly vegetatively reproducing species despite its capacity to occasionally produce some independent dispersal spores, such as thalloconidia. And *Umbilicaria muehlenbergii* with obvious ascomata on its upper surface, is widely accepted as a strictly sexually reproducing species.

### Distinguishable clades in *Trebouxia*

The species delimitations in the green algae genus *Trebouxia* are relatively poorly understood and many undescribed lineages have been detected by molecular methods [43]. It has been reported that the morphospecies *Trebouxia jamesii* contains some phylogenetic species [44–46]. It has been revealed that some identical genotypes of *T. jamesii* are shared among different lichen species, and the ITS phylogeny did not support the conclusion that *T. jamesii* subsp. *jamesii* and *T. jamesii* subsp. *angustilobata* were conspecific [47]. For this reason, phylogenetic analyses (Fig. 3) and DNA barcoding (Additional file 2: Figure S1) based on algal ITS sequences were executed to distinguish *Trebouxia* lineages in the present study. However, there were two gaps (5.0–5.5 %, 9.5–11 %) as shown in Additional file 2: Figure S1. A threshold of 5.5 % of ITS sequence variations was used to distinguish closely related *Trebouxia* species because the ITS variance among algal specieswas generally less than about 8 % [48]. Consequently, three undescribed lineages (TsA, TsB, and TsC) were identified and the traditional *T. jamesii* species was treated as two separate groups (TjA and TjB) using the 5.5 % variantion in ITS region threshold.

### Impact of ecogeographic factors on the diversity of mycobionts and photobionts of *Umbilicaria*

It has been demonstrated that ecogeographic factors can play a more important role in determining photobiont diversity than mycobiont haplotypes [49, 50]. Although no corresponding data are available at the species level, this study shows that ecological factors such as the atmospheric water (Fig. 1) supply may play an important role in shaping the distribution pattern of lichens on a large scale.

However, some studies have indicated that differential photobiont preferences in lichens cannot be explained on the spatial scale [12]. The photobionts in *Mastodia tessellata* from two locations far away (Chile and Australia) have been found sharing the same genotype [51]. Furthermore, some other factors, such as founder effects, may cause differences in the genetic diversity of lichens during long-distance colonization [52].

In the current study, samples were collected from six areas whose distances among them range from dozens to thousands of kilometers, so the climates in these sites are fairly different (Fig. 1). Though some haplotypes were found to be shared by individuals from different sites, the distributions of both symbionts appeared to be ecogeographically dependent. The most common haplotypes differed from collection site (Fig. 4, Additional file 2: Figure S2). However, in some cases, for example, the samples of *U. esculenta* from LC or those of *U. muehlenbergii* from YK, no dominant haplotypes were observed. The current study supports the conclusion that the ecogeographic factors play a more important role in determining photobiont diversity than mycobiont’s haplotypes and that it does so because the lichens with the same mycobiont haplotype harbored algal partners with different haplotypes if they were from different areas (Additional file 2: Figure S2). Nevertheless, some algal haplotypes were distributed across a broad range, which indicated that other factors would also contribute to the diversities of photobionts beyond ecological and geographic ones. Ecogeographic conditions appeared to be less influenced at the species level than at haplotype level.

### Reproductive strategy and mycobiont’s selectivity to photobionts

In general, the green algal photobionts in lichens are strictly asexual, but most mycobionts are still capable of using a sexual pathway. It has been suggested that sexual propagation plays an important role in long-distance dispersal of lichen-forming fungi [53, 54]. The clonality has its impact within a short distance [55]. Even if only one lichen-forming fungal ascospore finds a compatible photobiont after long-distance dispersal, a population could be still formedwithin a reasonable time frame through vegetative propagules [56]. For *Umbilicaria* lichens, a phylogenetic analysis of four *Umbilicaria* species across a long distance transect through Antarctica revealed that the *de novo* establishment of lichen symbiosis had occurred, and a low selectivity toward the photobiont was observed in the investigated species. This was interpreted as a strategy for survival under harsh Antarctic conditions [14].

In the present work, *Umbilicaria esculenta*, which disperses via vegetative structures, showed more variation in mycobionts than *U. muehlenbergii*, which disperses via sexual spores (Table 2). However, only one algal lineage, TjA, was found in *U. esculenta*. There were four types of photobionts, Tc, TjA, TjB, and Ts, in *U. muehlenbergii* (Additional file 2: Figure S2). The current study confirmed that the lichen, which had a sexual reproductive strategy, had less selectivity to its photosynthetic partners at the species level. The independent dispersal of the mycobiont (as ascospores) suggests that it has to decrease the selectivity to the compatible photobionts for re-lichenization shortly after the germination of ascospores. Otherwise, the ascospore will die, because it cannot obtain nutrients from photobionts. That means the lichen species with sexual reproductive strategies are apt to form new lichen thalli with a wide range of photobionts in both harsh and temperate environments. This shows that the reproductive strategy is the main reason for the mycobiont’s selectivity to photobionts, although the available photobionts are largely dependent on environmental conditions.

### Algal pool

*Trebouxia arboricola* was found to be a phycobiont partner in four bark-inhabiting lichen species without vegetative propagules. A free-living *T. arboricola* pool served as a source of potential photobionts [7]. The algal pool was confirmed in a further study. The photobiont diversity in a lichen community comprising nine saxicolous or chalcophilous species was investigated and the results indicated that each lichen species contained the same single algal species *T. jamesii* as photobiont partner [8].

Individuals of *U. esculenta* and *U. muehlenbergii* can share their photosynthetic partners with the lichens from the same sites, and some algal haplotypes can be found in different places (Fig. 4). However, *U. esculenta* shared its photobiont solely with *U. muehlenbergii. U. muehlenbergii* were found to share algal partners with a very broad range of other lichen, including those from *Boreoplaca* (Ophioparmaceae, Umbilicariales) and *Xanthoparmelia* (Parmeliaceae, Lecanorales) whose phylogenetic positions are far from those of Umbilicariales. The current results indicated that there existed an algal pool, especially in YK. Neighboring thalli form *Boreoplaca*, *Candelaria*, *Parmelia*, *Rhizoplaca*, *Umbilicaria*, and *Xanthoparmelia* in YK were examined, and four different *Trebouxia* lineages (Tc, Ti, TsB, and TsC) were shown to occur in different lichen species (Fig. 4). However, it seems that only lichens with sexual reproductive strategies can take in compatible algae from this pool.

### Reproductive strategy and diversity of lichen symbionts

The AMOVA analysis showed the genetic variation of photobiont from *U. esculenta* to be more influenced by local environments than *U. muehlenbergii* (46.98:18.00). Only TjA, which was the sole algal species in *U. esculenta* was considered for *U. muehlenbergii*, the same results were obtained (Table 1). This suggested a large amount of variation among photobionts in *U. muehlenbergii* in one locality. This considerable amount of variation could be explained by different algal species in this sexually reproducing lichen. The AMOVA analysis indicated that geographic factors played a less important role in structuring photobiont diversity in lichens with sexual strategy and that this was because these lichen-forming fungi can associate with a wide range of photosynthetic partners.

Both gene diversity and nucleotide diversity can be used to show the genetic variability within the population of lichen at the haplotype level and nucleotide level [57–61]. The number of haplotypes for each mycobiont belonging to the Umbilicariales was low in the current study. However, of 99 and 63 algal ITS sequences from *U. esculenta* and *U. muehlenbergii*, a total of 10 and 23 algal haplotypes were present for them, respectively (Table 2), and the ratios between number of haplotypes for photobiont and that for mycobiont were 2.5 for *U. esculenta*, 11.5 for *U. muehlenbergii* (This value is 11 for *B. ultrafrigida* which also reproduce sexually). These results clearly indicated that sexual lichen harbors many more types of photobionts than asexual ones at haplotype level.

It has been reported that even in populations with vegetative propagation strategies, mycobionts whose haplotypes are the same also can associate with more than one photobiont genotype [11]. This phenomenon was also observed in *U. esculenta* (Additional file 2: Figure S2). A recent study suggested that vegetative propagation and somatic mutations and recombination could shape generic structure of lichens, and the recombination of lichen-forming fungi occurred even among some mostly vegetative reproducing lichen species [55]. In the current study, four kinds of mycobionts in *U. esculenta* associated with 10 haplotypes of TjA, resulting in 19 genotypes of the thalli, and some genotype combination which included haplotypes of both mycobionts and photobionts for the whole lichen, such as UeI-TjA1 and UeI-TjA17, are obviously abundant in different collecting sites. This indicates that the reproductive mode for *U. esculenta* is clonal and some genotypes for this lichen show local adaption so that they are dominant. It has also been proposed that, in a vegetative reproducing fungal population, highly fit symbiont combinations may execute successive selective sweeps. This particular combination of mycobionts and photobionts may expand in numbers and sweep through the population [62]. The absolute predominance genotypes for *U. esculenta* in YK and SC indicates that selective sweeps occurred in the evolutionary history and maybe an important factor in shaping the population structure.

## Conclusions

It has been revealed that genetic differentiation of lichen photobionts can be shaped by ecological and geographic factors. The current study shows that reproductive strategy also influences the population structure of lichens. Irrespective of whether *U. esculenta* or *U. muehlenbergii* differed significantly in nucleotide diversity, the variance between their genotype diversity (the combinations of haplotypes of both symbionts) tended to be reduced. The current study was the first to show that both sexual and vegetative reproduction can allow lichens to generate almost the same amount of diversity to adapt to their environments.

Recent studies have suggested that geographical variance or stressing conditions can cause the changes in the reproductive strategies of lichens [63, 64]. In this way, further work is needed to show whether strategy is a truly independent variable in shaping population genetic structures for lichens.

## Additional files

Additional file 1: Table S1. Haplotypes information of *Umbilicaria* and other six lichen genera across six sites. **Table S2.** Sequences retrieved from Genbank. **Table S3.** Primers used in this study. (DOC 285 kb) Additional file 2: Figure S1. Intraspecific and interspecific distances in *Trebouxia* species. **Figure S2.** The column diagram of mycobiont-photobiont haplotypes distribution. (DOCX 1428 kb)
