# Supplementary material for: Distribution patterns of haplotypes for symbionts from Umbilicaria esculenta and U. muehlenbergii reflect the importance of reproductive strategy in shaping population genetic structure
Source: BMC Microbiol. 2015 Oct 15;15:212. doi: 10.1186/s12866-015-0527-0 (PMC4608304; doi:10.1186/s12866-015-0527-0)
Supplement: Additional file 1: Table S1. — Haplotypes information of Umbilicaria and other six lichen genera across six sites. Table S2. Sequences retrieved from Genbank. Table S3. Primers used in this study. (DOC 285 kb) [file 12866_2015_527_MOESM1_ESM.doc]

**Table S1. Samples' information. The haplotypes in mycobionts are marked with Roman numerals; and he haplotypes of photobionts are numbered with Arabic numerals.**

*Unique algal haplotypes for those retrieved from NCBI such as *Boreoplaca ultrafrigida* (for example, TjA14, TjB4，TsB2 etc.), are not summarized in this table.

**The same GenBank accession number represents the identical sequences of these samples.

***The different symbols before the Sampling locality represent the collecting sites, which are the same as those in Figure 1(■:DR, Daxinganling Region, ■:HB, Hulun Buir, ■:LC, Liuan City, ■:SC, Shiyan City, ■:TC, Tonghua City, ■:YK, Yanbian Korean Autonomous Prefecture).

| species |  | Sampling locality | Sample number | Haplotype of fungus | Haplotype of algae^*^ | Genbank Accession Numbers | |
| --- | --- | --- | --- | --- | --- | --- | --- |
|  |  |  |  |  |  | fungal ITS^**^ | algal ITS |
| *Candelaria fibrosa* | ■^***^ | YK | YK14043 | CfI | Ti2 | KP226208 | KP226273 |
| *Candelariella* *coralliza* | ■ | YK | YK140035 | CcI | Ti1 | KP226207 | KP226274 |
| *Parmelia omphalodes* | ■ | YK | YK1404 | PoI | TsB1 | KP226214 | KP226275 |
|  | ■ | YK | YK140827 | PoI | TsB1 | KP226214 | KP226275 |
|  | ■ | YK | YK140205 | PoI | TsB1 | KP226214 | KP226275 |
| *P. squarrosa* | ■ | YK | YK140204 | PsI | TsC1 | KP226213 | KP226276 |
|  | ■ | YK | YK140210 | PsI | TsC2 | KP226213 | KP226276 |
| *Rhizoplaca* *subdiscrepans* | ■ | YK | YK140037 | RsI | Ti1 | KP226212 | KP226277 |
|  | ■ | YK | YK140039 | RsI | Ti1 | KP226212 | KP226277 |
| *Umbilicaria esculenta* | ■ | YK | YK14011 | UeI | TjA1 | KP226192 | KP226215 |
|  | ■ | YK | YK14012 | UeI | TjA1 | KP226192 | KP226215 |
|  | ■ | YK | YK14014 | UeI | TjA1 | KP226192 | KP226215 |
|  | ■ | YK | YK14016 | UeI | TjA1 | KP226192 | KP226215 |
|  | ■ | YK | YK14017 | UeI | TjA1 | KP226192 | KP226215 |
|  | ■ | YK | YK14018 | UeI | TjA1 | KP226192 | KP226215 |
|  | ■ | YK | YK14019 | UeI | TjA1 | KP226192 | KP226215 |
|  | ■ | YK | YK14020 | UeI | TjA1 | KP226192 | KP226215 |
|  | ■ | YK | YK14021 | UeI | TjA1 | KP226192 | KP226215 |
|  | ■ | YK | YK14022 | UeI | TjA1 | KP226192 | KP226215 |
|  | ■ | YK | YK14024 | UeI | TjA1 | KP226192 | KP226215 |
|  | ■ | YK | YK14025 | UeI | TjA1 | KP226192 | KP226215 |
|  | ■ | YK | YK14026 | UeI | TjA1 | KP226192 | KP226215 |
|  | ■ | YK | YK14027 | UeI | TjA1 | KP226192 | KP226215 |
|  | ■ | YK | YK14028 | UeI | TjA1 | KP226192 | KP226215 |
|  | ■ | YK | YK14029 | UeI | TjA1 | KP226192 | KP226215 |
|  | ■ | YK | YK14031 | UeI | TjA1 | KP226192 | KP226215 |
|  | ■ | YK | YK14033 | UeI | TjA1 | KP226192 | KP226215 |
|  | ■ | YK | YK14034 | UeI | TjA1 | KP226192 | KP226215 |
|  | ■ | YK | YK14035 | UeI | TjA1 | KP226192 | KP226215 |
|  | ■ | YK | YK14036 | UeI | TjA1 | KP226192 | KP226215 |
|  | ■ | YK | YK14037 | UeI | TjA1 | KP226192 | KP226215 |
|  | ■ | YK | YK14038 | UeI | TjA1 | KP226192 | KP226215 |
|  | ■ | YK | YK14040 | UeI | TjA1 | KP226192 | KP226215 |
|  | ■ | YK | YK14060 | UeI | TjA1 | KP226192 | KP226215 |
|  | ■ | YK | YK14064 | UeI | TjA1 | KP226192 | KP226215 |
|  | ■ | YK | YK14065 | UeI | TjA1 | KP226192 | KP226215 |
|  | ■ | YK | YK14066 | UeI | TjA1 | KP226192 | KP226215 |
|  | ■ | YK | YK14067 | UeI | TjA1 | KP226192 | KP226215 |
|  | ■ | YK | YK14071 | UeI | TjA1 | KP226192 | KP226215 |
|  | ■ | YK | YK14073 | UeI | TjA1 | KP226192 | KP226215 |
|  | ■ | YK | YK14074 | UeI | TjA1 | KP226192 | KP226215 |
|  | ■ | YK | YK14079 | UeI | TjA1 | KP226192 | KP226215 |
|  | ■ | YK | YK140-21 | UeI | TjA1 | KP226192 | KP226215 |
|  | ■ | YK | YK140-25 | UeI | TjA1 | KP226192 | KP226215 |
|  | ■ | YK | YK140-11 | UeI | TjA1 | KP226192 | KP226215 |
|  | ■ | YK | YK140-13 | UeI | TjA1 | KP226192 | KP226215 |
|  | ■ | YK | YK140-15 | UeI | TjA1 | KP226192 | KP226215 |
|  | ■ | YK | YK140-51 | UeI | TjA1 | KP226192 | KP226215 |
|  | ■ | YK | YK140-53 | UeI | TjA1 | KP226192 | KP226215 |
|  | ■ | YK | YK140-I21 | UeI | TjA1 | KP226192 | KP226215 |
|  | ■ | YK | YK140-I23 | UeI | TjA1 | KP226192 | KP226215 |
|  | ■ | YK | YK140-33 | UeI | TjA6 | KP226192 | KP226216 |
|  | ■ | YK | YK14013 | UeII | TjA1 | KP226196 | KP226217 |
|  | ■ | YK | YK14023 | UeII | TjA1 | KP226196 | KP226217 |
|  | ■ | YK | YK14039 | UeII | TjA1 | KP226196 | KP226217 |
|  | ■ | YK | YK14061 | UeII | TjA1 | KP226196 | KP226217 |
|  | ■ | YK | YK14063 | UeII | TjA1 | KP226196 | KP226217 |
|  | ■ | YK | YK14068 | UeII | TjA1 | KP226196 | KP226217 |
|  | ■ | YK | YK140-50 | UeII | TjA6 | KP226196 | KP226218 |
|  | ■ | YK | YK14058 | UeIII | TjA1 | KP226197 | KP226219 |
|  | ■ | YK | YK140-02 | UeIII | TjA3 | KP226197 | KP226220 |
|  | ■ | YK | YK140-0952 | UeIII | TjA1 | KP226197 | KP226221 |
|  | ■ | YK | YK14030 | UeIV | TjA1 | KP226198 | KP226222 |
|  | ■ | YK | YK14075 | UeIV | TjA1 | KP226198 | KP226222 |
|  | ■ | YK | YK14076 | UeIV | TjA1 | KP226198 | KP226222 |
|  | ■ | YK | YK14077 | UeIV | TjA1 | KP226198 | KP226222 |
|  | ■ | YK | YK140-021 | UeIV | TjA3 | KP226198 | KP226223 |
|  | ■ | YK | YK140-023 | UeIV | TjA3 | KP226198 | KP226223 |
|  | ■ | YK | YK140-024 | UeIV | TjA3 | KP226198 | KP226223 |
|  | ■ | YK | YK140-026 | UeIV | TjA3 | KP226198 | KP226223 |
|  | ■ | YK | YK140-027 | UeIV | TjA3 | KP226198 | KP226223 |
|  | ■ | YK | YK140-036 | UeIV | TjA3 | KP226198 | KP226223 |
|  | ■ | YK | YK140-0944 | UeIV | TjA1 | KP226198 | KP226224 |
|  | ■ | YK | YK140-090 | UeIV | TjA3 | KP226198 | KP226225 |
|  | ■ | YK | YK140-091 | UeIV | TjA3 | KP226198 | KP226225 |
|  | ■ | YK | YK140-092 | UeIV | TjA3 | KP226198 | KP226225 |
|  | ■ | LC | LC1401 | UeI | TjA1 | KP226194 | KP226226 |
|  | ■ | LC | LC1405 | UeI | TjA6 | KP226194 | KP226227 |
|  | ■ | LC | LC1406 | UeI | TjA9 | KP226194 | KP226228 |
|  | ■ | LC | LC1409 | UeI | TjA4 | KP226194 | KP226229 |
|  | ■ | LC | LC14010 | UeI | TjA4 | KP226194 | KP226229 |
|  | ■ | LC | LC14012 | UeI | TjA6 | KP226194 | KP226227 |
|  | ■ | LC | LC14020 | UeI | TjA3 | KP226194 | KP226230 |
|  | ■ | LC | LC1403 | UeIV | TjA4 | KP226199 | KP226231 |
|  | ■ | LC | LC14013 | UeIV | TjA6 | KP226199 | KP226232 |
|  | ■ | LC | LC14014 | UeIV | TjA7 | KP226199 | KP226233 |
|  | ■ | LC | LC14015 | UeIV | TjA4 | KP226199 | KP226231 |
|  | ■ | LC | LC14017 | UeIV | TjA3 | KP226199 | KP226234 |
|  | ■ | LC | LC14018 | UeIV | TjA7 | KP226199 | KP226233 |
|  | ■ | LC | LC14019 | UeIV | TjA7 | KP226199 | KP226233 |
|  | ■ | SC | SC1401 | UeI | TjA7 | KP226193 | KP226235 |
|  | ■ | SC | SC1403 | UeI | TjA17 | KP226193 | KP226236 |
|  | ■ | SC | SC1404 | UeI | TjA10 | KP226193 | KP226237 |
|  | ■ | SC | SC1406 | UeI | TjA8 | KP226193 | KP226238 |
|  | ■ | SC | SC1407 | UeI | TjA17 | KP226193 | KP226236 |
|  | ■ | SC | SC1409 | UeI | TjA17 | KP226193 | KP226236 |
|  | ■ | SC | SC14010 | UeI | TjA17 | KP226193 | KP226236 |
|  | ■ | SC | SC14012 | UeI | TjA2 | KP226193 | KP226239 |
|  | ■ | SC | SC14015 | UeI | TjA17 | KP226193 | KP226236 |
|  | ■ | SC | SC14016 | UeI | TjA17 | KP226193 | KP226236 |
|  | ■ | SC | SC14018 | UeI | TjA17 | KP226193 | KP226236 |
|  | ■ | SC | SC14019 | UeI | TjA7 | KP226193 | KP226235 |
|  | ■ | SC | SC14020 | UeI | TjA17 | KP226193 | KP226236 |
|  | ■ | TC | TC140317 | UeI | TjA4 | KP226195 | KP226240 |
|  | ■ | TC | TC140019 | UeIV | TjA4 | KP226200 | KP226240 |
|  | ■ | TC | TC140020 | UeIV | TjA4 | KP226200 | KP226240 |
|  | ■ | TC | TC1403 | UeIV | TjA1 | KP226200 | KP226241 |
|  | ■ | TC | TC14013 | UeIV | TjA1 | KP226200 | KP226241 |
| *U. muehlenbergii* | ■ | YK | YK140-043 | UmI | TjA3 | KP226205 | KP226242 |
|  | ■ | YK | YK140-044 | UmI | TjA13 | KP226205 | KP226243 |
|  | ■ | YK | YK140-001 | UmI | Tc1 | KP226205 | KP226244 |
|  | ■ | YK | YK140-032 | UmII | TjB3 | KP226202 | KP226245 |
|  | ■ | YK | YK140-047 | UmII | TjA7 | KP226202 | KP226246 |
|  | ■ | DR | DR1401 | UmI | TjA16 | KP226206 | KP226247 |
|  | ■ | DR | DR1402 | UmI | TjA16 | KP226206 | KP226247 |
|  | ■ | DR | DR1403 | UmI | TjA16 | KP226206 | KP226247 |
|  | ■ | DR | DR1404 | UmI | TsA3 | KP226206 | KP226248 |
|  | ■ | DR | DR1406 | UmI | TjA18 | KP226206 | KP226249 |
|  | ■ | DR | DR1407 | UmI | TjA16 | KP226206 | KP226247 |
|  | ■ | DR | DR1408 | UmI | TjA15 | KP226206 | KP226250 |
|  | ■ | DR | DR1409 | UmI | TjA15 | KP226206 | KP226250 |
|  | ■ | DR | DR14010 | UmI | TsA3 | KP226206 | KP226248 |
|  | ■ | DR | DR14011 | UmI | TjA16 | KP226206 | KP226247 |
|  | ■ | DR | DR14012 | UmI | TjA15 | KP226206 | KP226250 |
|  | ■ | DR | DR14013 | UmI | TsA3 | KP226206 | KP226248 |
|  | ■ | DR | DR14014 | UmI | TjA13 | KP226206 | KP226251 |
|  | ■ | DR | DR14015 | UmI | TjA18 | KP226206 | KP226249 |
|  | ■ | DR | DR14018 | UmI | TjA16 | KP226206 | KP226247 |
|  | ■ | DR | DR14020 | UmI | TsA3 | KP226206 | KP226248 |
|  | ■ | DR | DR14016 | UmII | TjA11 | KP226201 | KP226252 |
|  | ■ | DR | DR14017 | UmII | TsA3 | KP226201 | KP226253 |
|  | ■ | DR | DR14021 | UmII | TjA11 | KP226201 | KP226254 |
|  | ■ | HB | HB140a6 | UmI | TjB2 | KP226203 | KP226255 |
|  | ■ | HB | HB140c1 | UmI | TjB1 | KP226203 | KP226256 |
|  | ■ | HB | HB140p1 | UmI | TjB7 | KP226203 | KP226257 |
|  | ■ | HB | HB140p2 | UmI | TjA13 | KP226203 | KP226258 |
|  | ■ | HB | HB140p3 | UmI | TjA16 | KP226203 | KP226259 |
|  | ■ | HB | HB140p4 | UmI | TjA10 | KP226203 | KP226260 |
|  | ■ | HB | HB140p5 | UmI | TjB2 | KP226203 | KP226255 |
|  | ■ | HB | HB140p8 | UmI | TjB7 | KP226203 | KP226257 |
|  | ■ | HB | HB140p9 | UmI | TjB7 | KP226203 | KP226257 |
|  | ■ | HB | HB140p10 | UmI | TjB7 | KP226203 | KP226257 |
|  | ■ | HB | HB140x2-2 | UmI | TjB2 | KP226203 | KP226255 |
|  | ■ | HB | HB140x12 | UmI | TjB2 | KP226203 | KP226255 |
|  | ■ | HB | HB140x13 | UmI | TjB7 | KP226203 | KP226257 |
|  | ■ | HB | HB140x14 | UmI | TjB6 | KP226203 | KP226261 |
|  | ■ | HB | HB140x15 | UmI | TjB2 | KP226203 | KP226255 |
|  | ■ | HB | HB140x17 | UmI | TjB2 | KP226203 | KP226255 |
|  | ■ | HB | HB140x18 | UmI | TjA18 | KP226203 | KP226262 |
|  | ■ | HB | HB140x19 | UmI | TjA18 | KP226203 | KP226262 |
|  | ■ | HB | HB140x20 | UmI | TjB2 | KP226203 | KP226255 |
|  | ■ | HB | HB140x21 | UmI | TjB2 | KP226203 | KP226255 |
|  | ■ | HB | HB140x22 | UmI | TjB8 | KP226203 | KP226263 |
|  | ■ | HB | HB140x23 | UmI | TjA12 | KP226203 | KP226264 |
|  | ■ | HB | HB140x24 | UmI | TjA15 | KP226203 | KP226265 |
|  | ■ | HB | HB140x26 | UmI | TjA13 | KP226203 | KP226258 |
|  | ■ | HB | HB140x27 | UmI | TjA12 | KP226203 | KP226264 |
|  | ■ | HB | HB140x29 | UmI | TjA19 | KP226203 | KP226266 |
|  | ■ | HB | HB140xa2 | UmI | TjB2 | KP226203 | KP226255 |
|  | ■ | HB | HB140xa6 | UmI | TjB2 | KP226203 | KP226255 |
|  | ■ | HB | HB140xa9-1 | UmI | TjB2 | KP226203 | KP226255 |
|  | ■ | HB | HB140xa10 | UmI | TjB5 | KP226203 | KP226267 |
|  | ■ | HB | HB140p11 | UmII | TjA21 | KP249926 | KP226268 |
|  | ■ | HB | HB140x1-1 | UmII | TjA22 | KP249926 | KP226269 |
|  | ■ | HB | HB140x28 | UmII | TjA20 | KP249926 | KP226270 |
|  | ■ | TC | TC14011 | UmI | TjA5 | KP226204 | KP226271 |
|  | ■ | TC | TC1405 | UmI | TjA5 | KP226204 | KP226271 |
|  | ■ | TC | TC1407 | UmI | TjA13 | KP226204 | KP226272 |
|  | ■ | TC | TC14072 | UmI | TjA5 | KP226204 | KP226271 |
|  | ■ | TC | TC140013 | UmI | TjA5 | KP226204 | KP226271 |
|  | ■ | TC | TC14017 | UmI | TjA5 | KP226204 | KP226271 |
| *Xanthoparmelia conspersa* | ■ | YK | YK140-2 | XcI | Tc1 | KP226211 | KP226278 |

**Table S2.** Sequences retrieved from Genbank

| **Algal ITS rDNA Sequences retrieved from Genbank** | | | |
| --- | --- | --- | --- |
| Species name | GenBank No. | The systematic position |  |
| *Dictyochloropsis reticulate* | FJ936174 | Chlorophyta; Trebouxiophyceae |  |
| *Koliella longiseta* | AJ431677 | Chlorophyta; Trebouxiophyceae |  |
| *Trebouxia* photobiont | EU715061 | Chlorophyta; Trebouxiophyceae |  |
| *Trebouxia corticola* | AB177831 | Chlorophyta; Trebouxiophyceae |  |
| *T. gelatinosa* | AJ249568 | Chlorophyta; Trebouxiophyceae |  |
| *T. impressa* | AF345891 | Chlorophyta; Trebouxiophyceae |  |
| *T. jamesii* | AJ511354 | Chlorophyta; Trebouxiophyceae |  |
| *T. jamesii* | AJ249571 | Chlorophyta; Trebouxiophyceae |  |
| *T. jamesii* | AF128270 | Chlorophyta; Trebouxiophyceae |  |
| *T. jamesii* | FJ170733 | Chlorophyta; Trebouxiophyceae |  |
| *T. jamesii* | AF242457 | Chlorophyta; Trebouxiophyceae |  |
| Uncultured *Trebouxia* photobiont | EF432561 | Chlorophyta; Trebouxiophyceae |  |
| Uncultured *Trebouxia* photobiont | EU715059 | Chlorophyta; Trebouxiophyceae |  |
| Uncultured *Trebouxia* photobiont | AM159208 | Chlorophyta; Trebouxiophyceae |  |
| Uncultured *Trebouxia* photobiont | EU416220 | Chlorophyta; Trebouxiophyceae |  |
| **Fungal ITS rDNA Sequences retrieved from Genbank** | | |  |
| *Candelaria fibrosa* | EF535206 | Ascomycota; Candelariales; Candelariaceae |  |
| *Rhizoplaca subdiscrepans* | HM577232 | Ascomycota; Lecanorales; Lecanoraceae |  |
| *Xanthoparmelia conspersa* | DQ394369 | Ascomycota; Lecanorales; Parmeliaceae |  |

**Table S3.** Primers used in this study

| Premers | Sequences(5’→3’) | Amplied region | literature |
| --- | --- | --- | --- |
| nrSSU-1780-5’ | CTGCGGAAGGATCATTGATTC | Algal ITS rDNA | Piercey-Normore and Depriest 2001 |
| nrLSU-0012-3’ | AGTTCAGCGGGTGGTCTTG | Algal ITS rDNA | Piercey-Normore and Depriest 2001 |
| All500bf | GATGCATTCAACGAGCCTA | Algal ITS rDNA | Helms *et al*. 2001 |
| LR3 | CCGTGTTTCAAGACGGG | Algal ITS rDNA | Helms *et al*. 2001 |
| ITS1T | GGAAGGATCATTGAATCTATCGT | Algal ITS rDNA | Kroken & Taylor 2000 |
| ITS4T | GGTTCGCTCGCCGCTACTA | Algal ITS rDNA | Kroken & Taylor 2000 |
| ITS5 | GGAAGTAAAAGTCGTAACAAGG | Fungal ITS rDNA | White *et al*. 1990 |
| ITS1 | TCCGTAGGTGAACCTGCGG | Fungal ITS rDNA | White *et al*. 1990 |
| ITS1F | CTTGGTCATTTAGAGGAAGTAA | Fungal ITS rDNA | Gardes & Bruns 1993 |
| ITS4 | TCCTCCGCTTATTGATATGC | Fungal ITS rDNA | White *et al*. 1990 |
