# Supplementary material for: Distribution patterns of haplotypes for symbionts from Umbilicaria esculenta and U. muehlenbergii reflect the importance of reproductive strategy in shaping population genetic structure
Source: BMC Microbiol. 2015 Oct 15;15:212. doi: 10.1186/s12866-015-0527-0 (PMC4608304; doi:10.1186/s12866-015-0527-0)
Supplement: Additional file 2: Figure S1. — Intraspecific and interspecific distances in Trebouxia species. Figure S2. The column diagram of mycobiont-photobiont haplotypes distribution. (DOCX 1428 kb) [file 12866_2015_527_MOESM2_ESM.docx]

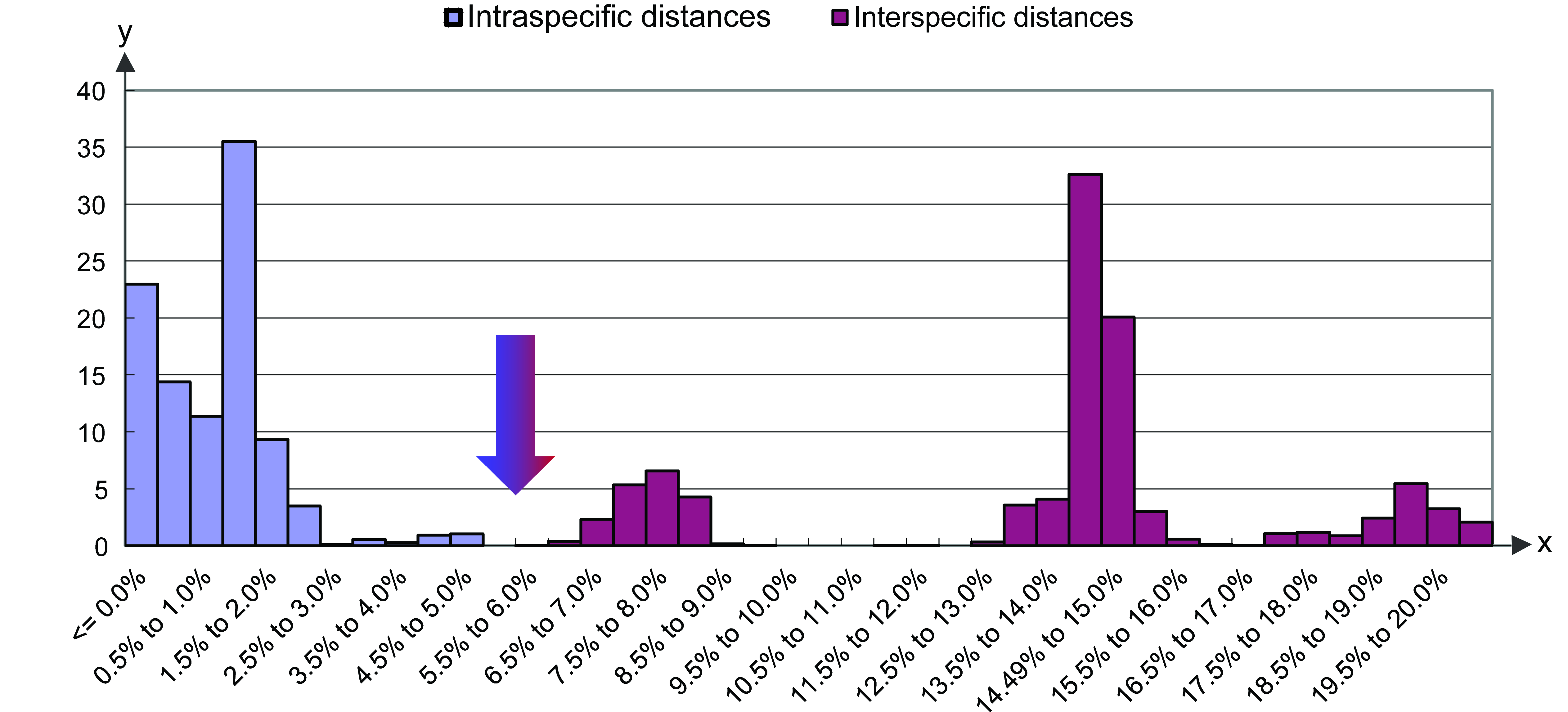


**Additional file2: Figure S1**. Intraspecific and interspecific distances in *Trebouxia* species. The degrees of variations and the percentage of individuals are illustrated on the X- and Y- axes respectively. It is 5.5% in our study that can be looked as the threshold distinguishing different species.

**
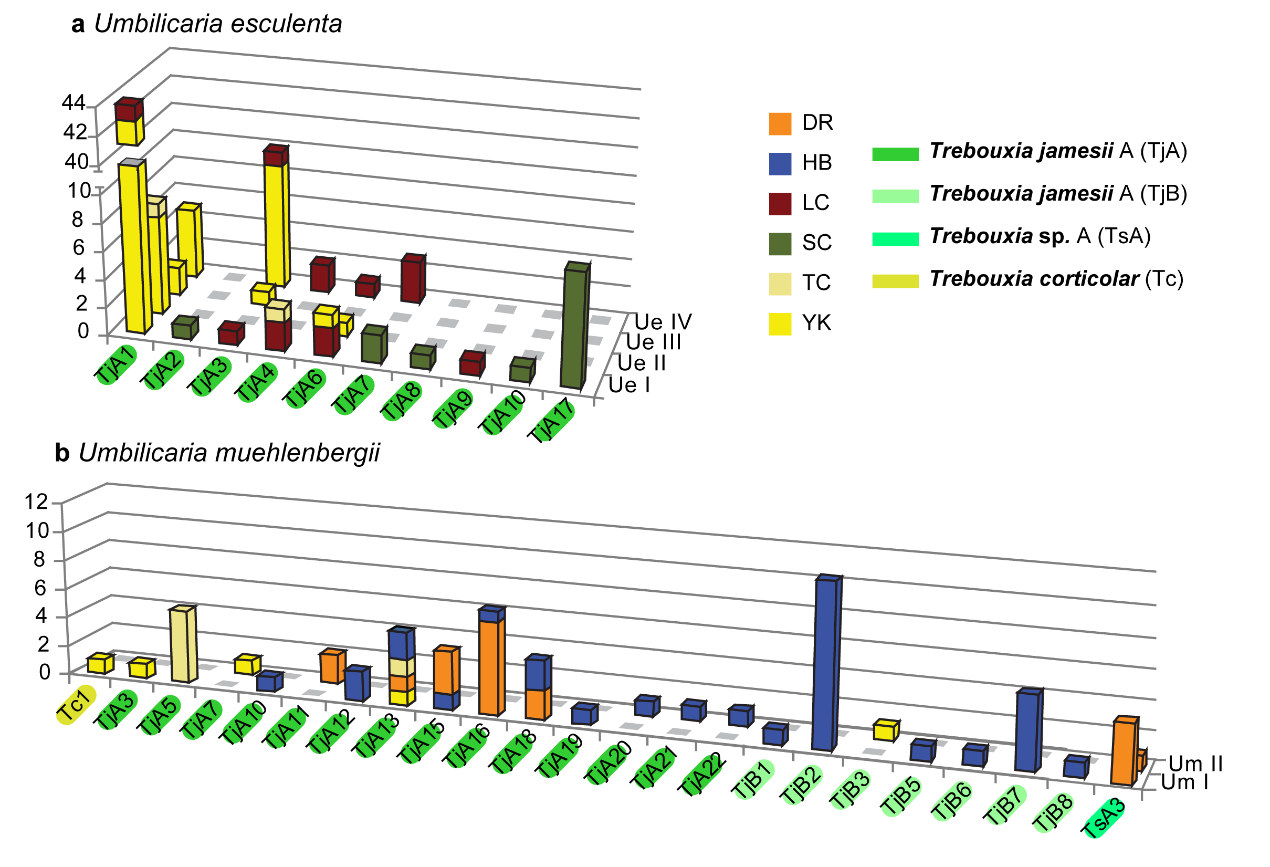
**

**Additional file2: Figure S2**. The column diagram of mycobiont-photobiont haplotypes distribution. The color of the column indicates different sampling localities, and the height of the column illustrates the numbers of individuals with the same myco-photobiont genotype.
